# Past hurts, present hurdles: Analyzing the impact of adult socioeconomic status and healthcare access on cancer screening among childhood abuse survivors

**DOI:** 10.1371/journal.pgph.0007073

**Published:** 2026-08-26

**Authors:** Qaidar Alizai, Azza Sarfraz, Meher Angez, Areesh Mevawalla, Odysseas P. Chatzipanagiotou, Abdulaziz Elemosho, Rida Ejaz, Kizuki Yuza, Sebastian Ekenze, Timothy M. Pawlik

**Affiliations:** Department of Surgery, The Ohio State University Wexner Medical Center and James Comprehensive Cancer Center, Columbus, Ohio, United States of America; Université de Sherbrooke: Universite de Sherbrooke, CANADA

## Abstract

Childhood abuse is associated with long-term health consequences and may influence uptake of preventive healthcare. We evaluated whether adult socioeconomic experiences and healthcare access modify the association between childhood abuse and adherence to U.S. Preventive Services Task Force (USPSTF) cancer screening guidelines. We analyzed 2020 Behavioral Risk Factor Surveillance System (BRFSS) data among respondents who answered the childhood abuse item and were eligible for colorectal (CRC; 50–74years), cervical (women 25–64years, excluding hysterectomy), and/or breast screening (women 40–74years), adapted to BRFSS 5-year age categories. Screening adherence was compared by abuse history. Survey-weighted Poisson regression estimated multivar incidence rate ratios (aIRRs), and stratified models assessed heterogeneity across adult socioeconomic factors and healthcare access. Among 123,169 respondents (weighted U.S. adult population: 68.08 million), 43.7% reported childhood abuse. Eligible cohorts included 27,368 for cervical screening and 39,986 women for breast screening (ages 40–74). In fully adjusted survey-weighted models, childhood abuse was associated with modestly lower adherence to cervical (aIRR = 0.97, 95%CI 0.94–0.99) and breast screening (aIRR = 0.96, 95%CI 0.93–0.99). In stratified analyses, inverse associations for cervical screening persisted across multiple socioeconomic strata, including college graduates (aIRR = 0.96, 95%CI 0.94–0.98), employed respondents (aIRR = 0.97, 95%CI 0.95–0.98), and married respondents (aIRR = 0.97, 95%CI 0.96–0.99). For breast screening, childhood abuse was consistently associated with lower adherence across most strata, with stronger associations among divorced/widowed/separated respondents (aIRR = 0.92, 95%CI 0.90–0.95) and lower-income respondents (aIRR = 0.95, 95%CI 0.93–0.96), and a pronounced deficit among uninsured women (aIRR = 0.86, 95%CI 0.78–0.95). Childhood abuse was associated with modestly lower adherence to cervical and breast cancer screening (with smaller, less consistent differences for CRC), and these associations varied across socioeconomic and healthcare-access strata. These findings support targeted, trauma-informed strategies to improve screening uptake among survivors of childhood abuse, particularly in settings where structural barriers to preventive care persist.

## Introduction

Childhood abuse is a pervasive public health problem with long-term consequences for physical, sexual, and mental health [1]. Survivors of abuse are at increased risk of chronic diseases, mental health disorders, and unhealthy behaviors, all of which can contribute to poorer health outcomes in adulthood [2,3]. Importantly, emerging evidence also links childhood abuse to certain cancers, particularly cervical cancer, through pathways such as high-risk sexual exposures, chronic stress, immune dysregulation, and reduced engagement in preventive care [4,5]. Given that timely cancer screening is a cornerstone of early detection and survival, understanding how early adversity influences screening behaviors is critical.

Survivors of childhood abuse may be less likely to comply with recommended cancer screening guidelines, placing these individuals at risk of delayed diagnosis and worse outcomes [6–8]. This lower adherence has been attributed to a combination of psychosocial barriers, mistrust of healthcare providers, and socioeconomic disadvantage that often follows early adversity [6,8]. Physical, sexual, and emotional abuse may also increase the risk of unhealthy behaviors like cigarette smoking, substance abuse, and risky sexual practices [6,8]. In particular, sexual abuse may heighten discomfort with sexual health services, such as Pap testing or breast exams performed by male practitioners [6,9]. Beyond these psychosocial and behavioral factors, survivors often experience cumulative disadvantages across the life course [10]. Many individuals may have baseline limited socioeconomic resources, and the added impact of abuse on education, employment, and mental and physical health compounds barriers to care [3,11,12].

An important gap in the literature is whether adult socioeconomic status and healthcare access modify the association between childhood abuse and cancer screening. Even among individuals with similar abuse histories, differences in insurance coverage, usual source of care, cost barriers, education, and income may either buffer screening deficits or widen disparities. Clarifying how adult circumstances shape screening patterns is essential to identify high-risk subgroups and inform targeted interventions and policy. The aim of the current study was to define how socioeconomic status and healthcare access in adult life influence the association between childhood abuse and adherence to colorectal, cervical, and breast cancer screening guidelines.

## Materials and methods

### Data source and study population

Data from the 2020 Behavioral Risk Factor Surveillance System (BRFSS), a nationally representative telephone survey of U.S. adults conducted annually by the Centers for Disease Control and Prevention (CDC) was used [13]. BRFSS collects information on health behaviors, chronic conditions, and preventive services through structured interviews. Participants aged ≥18 years who answered “yes” or “no” to questions about childhood abuse in the Adverse Childhood Experiences (ACE) module were eligible. Physical abuse was defined as responding “once” or “more than once” to ACEHURT1: “*Not including spanking, before age 18, how often did a parent or adult in your home ever hit, beat, kick, or physically hurt you in any way?*” Emotional abuse was defined as a similar response to ACESWEAR: “*How often did a parent or adult in your home ever swear at you, insult you, or put you down?*” Sexual abuse was identified based on any affirmative response to the following items: (i) “*How often did anyone, at least 5 years older than you or an adult, ever touch you sexually?*” (ii) “*How often did anyone at least 5 years older than you or an adult try to make you touch them sexually?*” and/or (iii) “*How often did anyone at least 5 years older than you or an adult force you to have sex?*” Responses of “never” were coded as no abuse; respondents who answered: “don’t know,” “refused,” or had missing data for all three items were excluded. Individuals reporting at least one type of abuse were categorized as the “abuse” group, and individuals reporting none were categorized as “non-abuse” group [6].

Additional ACE items captured other household challenges, including parental separation/divorce, living with someone who abused alcohol or drugs, violence, mental illness, or incarceration [6,14]. The analytic cohort was further restricted to respondents eligible for colorectal, cervical, or breast cancer screening based on U.S. Preventive Services Task Force (USPSTF) guidelines:

- **Colorectal cancer (CRC):** Eligibility was defined as adults aged 50–74 years of either sex without a prior diagnosis of CRC (USPSTF 2016) [15].
- **Cervical cancer:** Eligibility was defined as women aged 25–64 years without a history of cervical cancer or hysterectomy (USPSTF 2018). Because BRFSS reports age in 5-year categories (with the youngest category 18–24 years), and since ages 21–24 cannot be isolated, age 25 years was used as the lower bound to avoid misclassifying women aged 18–20 years as eligible for cervical cancer screening [16].
- **Breast cancer:** Eligibility was defined as women aged 40–74 years without a prior diagnosis of breast cancer, consistent with the USPSTF 2016 recommendation for biennial screening in the routine screening age group [17]*.* Furthermore, to align with USPSTF age-based screening recommendations, additional sensitivity analysis was performed for breast screening adherence restricting eligibility to women aged 50–74 years.

Colorectal, cervical, and breast cancer screening were selected as these cancers could be consistently defined using core 2020 BRFSS variables among USPSTF-eligible populations with sufficient sample sizes. Furthermore, 2020 BRFSS was selected as it provided the largest ACE-available analytic sample among the most recent years allowing consistent assessment of ACE exposure and cancer screening outcomes.

This study used publicly available, de-identified data from the BRFSS. Data files are available at https://www.cdc.gov/brfss/annual_data/annual_data.htm and were accessed on 5^th^ August 2025 [13]. BRFSS participants provide informed consent at the time of the telephonic survey administration by the CDC [13]. Because this study was a secondary analysis of publicly available, de-identified BRFSS data and did not involve access to private, identifiable information, it was determined to be exempt from institutional review board approval by the Ohio State University Institutional Review Board (IRB ID: STUDY20261166) [13].

### Exposure, outcomes, and covariates

The primary exposure was self-reported childhood abuse, classified as any physical, emotional, or sexual abuse versus none, based on responses to the ACE module. The primary outcomes were adherence to USPSTF cancer screening recommendations. Among respondents eligible for colorectal cancer (CRC) screening, adherence was defined as reporting any of the following: colonoscopy within the past 10 years; sigmoidoscopy or CT colonography within the past 5 years; fecal immunochemical test (FIT) or fecal occult blood test (FOBT) within the past 1 year; or stool DNA testing within the past 1–3 years [15]. Among respondents eligible for cervical cancer screening, adherence was defined as a Pap test within the past 3 years, human papillomavirus (HPV) testing, or Pap plus HPV co-testing within the past 5 years, consistent with USPSTF guidance [16]. Similarly, among breast cancer screening eligible respondents, screening adherence was defined as history of a mammogram within the preceding 2 years [17]. Derivation of these guideline-based screening eligibility and adherence criteria from the BRFSS variables is detailed in **S1 Table**.

Covariates included demographic characteristics such as age (e.g., 18–34, 35–49, 50–64, 65–79, ≥ 80 years), sex (e.g., male, female), race (e.g., White, Black, Other), and ethnicity (e.g., Hispanic vs non-Hispanic). Socioeconomic indicators included education (e.g., college graduate vs less than college), employment status (e.g., employed; homemaker; unemployed, retired, or student), annual household income (<$75,000 vs ≥ $75,000), marital status (e.g., married; divorced, widowed, or separated; never married/unmarried couple) [18], and home ownership (own home vs rented/other) [19,20] Healthcare access variables included insurance type (e.g., uninsured, public, private), presence of a personal doctor, receipt of a routine health checkup (e.g., any checkup in prior 2 years vs checkup more than 2 years or never), and cost barriers, defined as reporting a need for healthcare in the past 12 months but being unable to obtain it due to expense [21].

Self-rated health status (e.g., fair/poor vs good/very good/excellent) and other non-abuse household challenges during childhood (e.g., parental separation or divorce, household substance use, violence, incarceration, or mental illness) were also assessed. In addition, adult socioeconomic factors (education, income, marital status, employment, and home ownership) and healthcare access measures were examined as potential effect modifiers of the association between childhood abuse and adherence to cancer screening guidelines.

### Statistical analyses

This was a cross-sectional study using between-subjects comparisons. Descriptive statistics for categorical variables were presented as weighted frequencies and percentages (%) and compared between participants with and without a history of childhood abuse using survey-weighted chi-square (χ²) tests. Continuous variables, where applicable, were summarized as medians with interquartile ranges (IQR) and compared using the Kruskal–Wallis test. Associations between childhood abuse and adherence to each USPSTF cancer screening guideline were examined separately using survey-weighted Poisson regression with robust variance. Results were reported as incidence rate ratios (IRRs) with 95% confidence intervals (CIs).

Multivariable regression models were adjusted for age, sex (for CRC models only), race/ethnicity, education, marital status, employment status, annual household income, home ownership, insurance type, rural/urban residence, prior history of skin or other cancers, self-rated health, and other non-abuse household challenges. Variance inflation factors were examined to assess multicollinearity among covariates; no significant collinearity was detected. Robust variance estimation was used to account for potential overdispersion and heteroscedasticity in the Poisson models. Given the categorical nature of the primary exposure and outcomes, no outlier exclusions were performed. Respondents with missing data on key variables were excluded from the relevant analyses using complete case analysis.

Stratified analyses assessed whether adult socioeconomic factors and healthcare access changed the association between abuse and screening. Subgroups included education level, annual household income, employment status, marital status, home ownership, insurance type, cost-related barriers to care, presence of a personal doctor, receipt of a routine checkup in the prior two years, and exposure to any other household challenges in childhood. Because multiple subgroup analyses were conducted, these findings were interpreted as hypothesis generating and emphasized effect sizes and confidence intervals over statistical significance, with subgroup estimates showing wide confidence intervals (reflecting smaller sample sizes) interpreted cautiously.

This was a secondary analysis of existing available BRFSS data; no sample size calculation was performed. The sample size was determined by the number of eligible respondents in the 2020 BRFSS who completed the ACE module for abuse. All analyses used BRFSS sampling weights, strata, and primary sampling units to account for the survey design. Two-sided P-values <0.05 were considered statistically significant. Analyses were conducted in Stata 18.0 (StataCorp, College Station, TX). Findings were reported in accordance with the STROBE (Strengthening the Reporting of Observational Studies in Epidemiology) checklist for cross-sectional studies (S1 File STROBE Checklist).

## Results

### Baseline characteristics

Among 123,169 respondents, representing a weighted U.S. adult population of 68.08 million, 56.3% [n = 69,398] reported no history of childhood abuse and 43.7% [n = 53,771] reported childhood abuse (Table 1, **S2 Table**). Overall, 52.3% [n = 67,669] were female and 62.7% [n = 92,548] identified as White. Self-rated health was fair or poor in 16.7% [n = 20,478] and a history of any cancer was reported by 12.4% [n = 21,614] of respondents.

**Table 1:** Baseline characteristics of respondents with and without a history of abuse.

Characteristic	Total (N = 123,169)	Non-abuse (N = 69,398)	Abuse (N = 53,771)	p-value
**Age groups, years**				**<0.001**
18–34	19,921 (27.3%)	9,648 (24.5%)	10,273 (30.7%)	
35–49	23,704 (22.9%)	11,991 (21.6%)	11,713 (24.4%)	
50–64	32,764 (25.4%)	17,594 (25.0%)	15,170 (26.0%)	
65–79	34,872 (18.3%)	21,634 (21.1%)	13,238 (14.9%)	
80+	11,908 (6.1%)	8,531 (7.8%)	3,377 (4.0%)	
**Sex**				0.546
Male	55,500 (47.7%)	31,323 (47.9%)	24,177 (47.3%)	
Female	67,669 (52.3%)	38,075 (52.1%)	29,594 (52.7%)	
**Race**				**<0.001**
White	92,548 (62.7%)	52,519 (62.0%)	40,029 (63.5%)	
Minority race	30,621 (37.3%)	16,879 (38.0%)	13,742 (36.5%)	
**Any cancer history**	21,614 (12.4%)	12,623 (12.8%)	8,991 (11.9%)	**<0.001**
**Any Household challenges**	58,334 (53.2%)	22,066 (36.6%)	36,268 (72.9%)	**<0.001**
**Education**				**<0.001**
College graduate	45,638 (26.0%)	26,420 (27.4%)	19,218 (24.2%)	
Less than college	77,531 (74.1%)	42,978 (72.6%)	34,553 (75.8%)	
**Employment status**				**<0.001**
Employed	60,912 (55.1%)	33,053 (53.6%)	27,859 (56.9%)	
Unemployed/Retired/Student	56,794 (39.3%)	33,155 (40.4%)	23,639 (38.0%)	
Homemaker	5,463 (5.6%)	3,190 (6.0%)	2,273 (5.1%)	
**Household income**				**<0.001**
Lower income (<$75,000)	67,148 (55.1%)	36,189 (52.9%)	30,959 (57.8%)	
Higher income (≥$75,000)	37,023 (29.2%)	21,136 (29.8%)	15,887 (28.6%)	
Missing/Unknown	18,998 (15.7%)	12,073 (17.4%)	6,925 (13.6%)	
**Marital status**				**<0.001**
Married	64,874 (50.7%)	38,192 (53.4%)	26,682 (47.5%)	
Divorced/Widowed/Separated	33,532 (21.4%)	18,965 (21.1%)	14,567 (21.8%)	
Never married	24,763 (27.9%)	12,241 (25.5%)	12,522 (30.8%)	
**Insurance type**				**<0.001**
Uninsured	11,185 (9.0%)	5,488 (7.9%)	5,697 (10.5%)	
Public insurance	8,950 (7.2%)	5,474 (7.8%)	3,476 (6.4%)	
Private insurance	9,115 (7.4%)	5,066 (7.3%)	4,049 (7.5%)	
Unknown/Missing	93,919 (76.2%)	53,370 (76.9%)	40,549 (75.4%)	

Compared with respondents without a history of childhood abuse, individuals with childhood abuse were younger (18–34 years: 24.5% [n = 9,648] vs. 30.7% [n = 10,273]) and more often reported household challenges (36.6% [n = 22,066] vs. 72.9% [n = 36,268]) (both p < 0.001; Table 1). These individuals were also less frequently married (53.4% [n = 38,192] vs. 47.5% [n = 26,682]), more often had household income < $75,000 (52.9% [n = 36,189] vs. 57.8% [n = 30,959]), and were more often uninsured (7.9%% [n = 5,488] vs. 10.5% [n = 5,697]). In addition, abuse survivors reported worse self-rated health (fair/poor: 14.3% [n = 9,911] vs. 19.7% [n = 10,567]) and were more often urban (80.2% [n = 55,671] vs. 83.2% [n = 44,750]), as well as more often reported cost barriers to care (8.7% [n = 4,410] vs. 17.4% [n = 7,144]) and were less likely to have a personal doctor (77.0% [n = 52,557] vs. 73.4% [n = 39,186]) (all p < 0.001; Table 1, **S1 Table**).

### Cancer screening adherence

Among the total analytic cohort, 58,390 respondents were eligible for CRC screening (no abuse: n = 33,088 vs. abuse: n = 25,302); 27,368 were eligible for cervical cancer screening (no abuse: n = 14,069 vs. abuse: n = 13,299); and 39,986 women were eligible for breast cancer screening (ages 40–74; no abuse: n = 21,922 vs. abuse: n = 18,064). Screening adherence was lower among respondents with childhood abuse across all three screening types: CRC 72.4% [n = 23,958] vs 71.9% [n = 18,205], cervical 83.9% [n = 11,797] vs. 81.6% [n = 10,848], and breast 73.0% [n = 15,998] vs. 67.0% [n = 12,100] (all p < 0.001; Table 2).

**Table 2:** Adherence to cancer screening among individuals with and without abuse history.

Screening type	Total	Non-abuse	Abuse	P-value
**CRC**	40,763 (26.2%)	23,270 (27.7%)	17,493 (24.4%)	**<0.001**
**Cervical cancer**	21,750 (79.6%)	11,346 (81.1%)	10,404 (78.1%)	**<0.001**
**Breast cancer**	28,098 (68.1%)	15,998 (70.9%)	12,100 (64.7%)	**<0.001**

### Adjusted analyses

In unadjusted Poisson regression, childhood abuse was associated with lower adherence to CRC (IRR 0.93 [95% CI, 0.90–0.96]), cervical (IRR 0.94 [95% CI, 0.91–0.97]), and breast (IRR 0.91 [95% CI, 0.88–0.94]) cancer screening (Table 3). In survey-weighted multivariable models, childhood abuse remained associated with lower adherence to CRC (aIRR 0.97 [95% CI, 0.94–1.00]), cervical (aIRR 0.97 [95% CI, 0.94–0.99]), and breast screening (aIRR 0.96 [95% CI, 0.93–0.99]). On additional sensitivity analysis that restricted the analytic cohort to breast screening eligibility among women aged 50–74 years, findings were consistent with the primary breast cancer screening model (aIRR 0.96 [95% CI, 0.93–0.99]) (Table 3).

**Table 3:** Association between childhood abuse and cancer screening adherence.

Cancer screening type	Univariate IRR (95% CI)	P-value	Multivariable IRR (95% CI)	P-value
CRC	0.93 (0.90-0.96)	**<0.001**	0.97 (0.94–1.00)	**0.022**
Cervical cancer	0.94 (0.91-0.97)	**<0.001**	0.97 (0.94–0.99)	**0.013**
Breast cancer	0.91 (0.88-0.94)	**<0.001**	0.96 (0.93–0.99)	**0.005**
**Sensitivity analyses**				
Breast cancer (age 50–74)	0.93 (0.90-0.96)	**<0.001**	0.96 (0.93-0.99)	**<0.001**

Across fully adjusted, survey-weighted models, insurance coverage was the dominant predictor of screening adherence. Relative to uninsured respondents, screening adherence was higher with public insurance (CRC aIRR 1.96 [1.74–2.20]; cervical aIRR 1.32 [1.22–1.43]; breast aIRR 1.73 [1.52–1.97]) and private insurance (CRC aIRR 1.83 [1.62–2.06]; cervical aIRR 1.32 [1.23–1.41]; breast aIRR 1.77 [1.56–2.00]; all p < 0.001). Higher income ≥ $75,000 (CRC aIRR 1.07 [1.04–1.11]; cervical aIRR 1.06 [1.03–1.09]; breast aIRR 1.06 [1.03–1.10]) and a college graduation (CRC aIRR 1.06 [1.03–1.09]; cervical aIRR 1.04 [1.02–1.07]; breast aIRR 1.06 [1.02–1.09]) were associated with modestly higher adherence, while minority status was associated with higher adherence than White respondents (CRC aIRR 1.06 [1.02–1.10]; cervical aIRR 1.05 [1.02–1.08]; breast aIRR 1.16 [1.12–1.20]). Rural residence was associated with slightly lower adherence (CRC aIRR 0.96 [0.93–0.99], p = 0.006; cervical aIRR 0.96 [0.93–1.00], p = 0.039; breast aIRR 0.97 [0.93–1.00], p = 0.081). After adjusting for other factors, childhood abuse remained independently associated with lower adherence for CRC (aIRR 0.97 [0.94–1.00], p = 0.022), cervical (aIRR 0.97 [0.94–0.99], p = 0.013), and breast screening (aIRR 0.96 [0.93–0.99], p = 0.005; **S3 Table**).

### Subgroup and effect modification analyses

**Fig 1:**
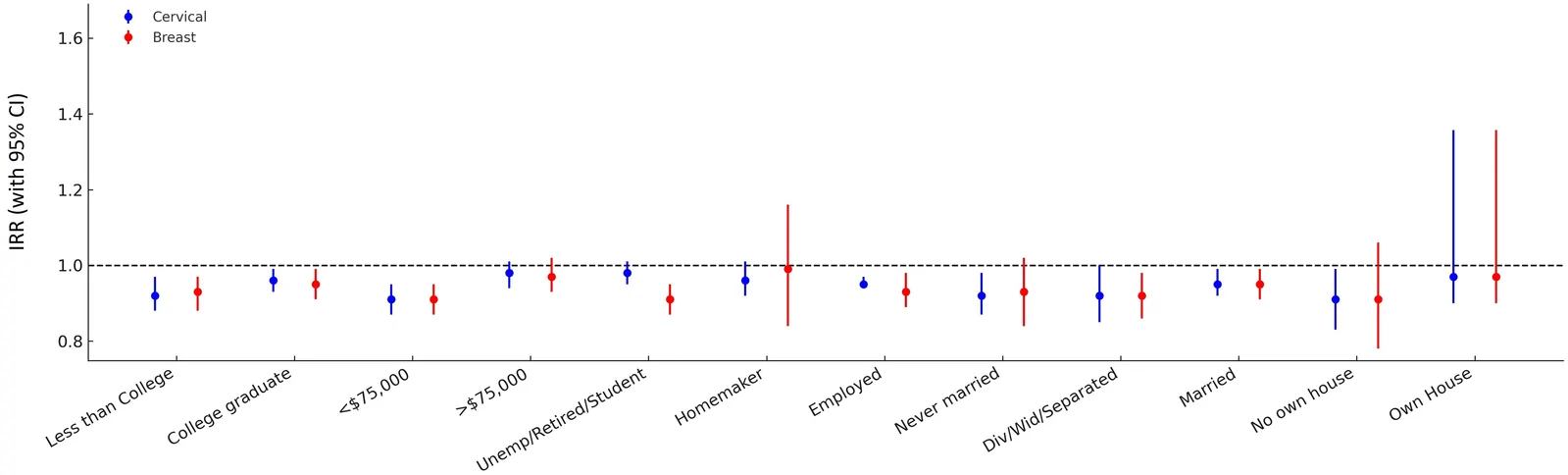
Effects of adult life socioeconomic characteristics on the association of childhood abuse with cervical and breast cancer screening.

**Fig 2:**
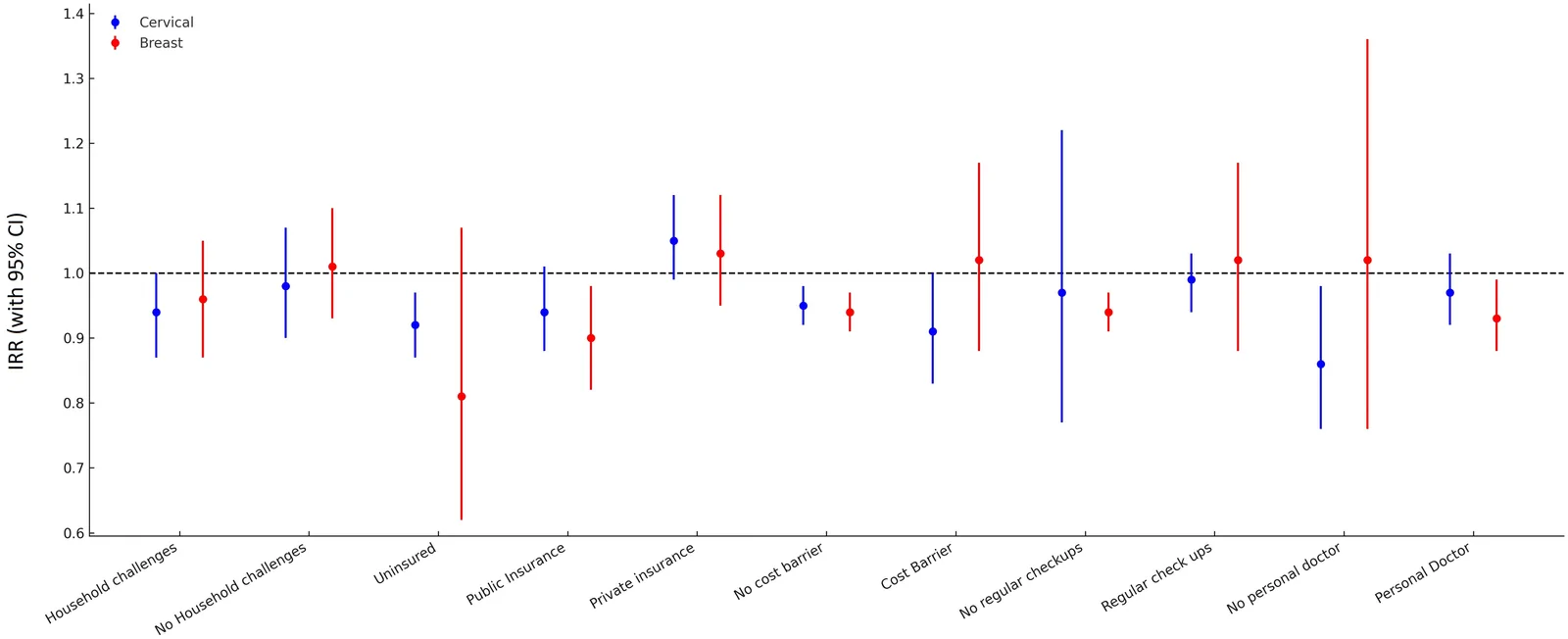
Effects of adult life healthcare access status on the association of childhood abuse with cervical and breast cancer screening.

In stratified analyses, the association between childhood abuse and CRC screening was small and frequently crossed the null across most strata, with one notable exception among individuals who were overdue for routine care (≥2 years/never: 1.11 [1.01–1.23]). In contrast, cervical screening had an inverse association across several strata (typical aIRR ~ 0.95–0.98), including college graduates (0.96 [0.94–0.98]), employed respondents (0.97 [0.95–0.98]), divorced/widowed/separated (0.95 [0.92–0.98]), and married respondents (0.97 [0.96–0.99]) (Table 4; Fig 1). For breast screening, childhood abuse was consistently associated with lower adherence across most strata, including lower income (0.95 [0.93–0.96]) and divorced/widowed/separated respondents (0.92 [0.90–0.95], the strongest inverse association). In access-stratified models, lower breast screening persisted among the uninsured (0.86 [0.78–0.95]) and individuals with public insurance (0.95 [0.91–1.00]), as well as among respondents with a routine checkup within 2 years (0.96 [0.95–0.97]), no cost barriers (0.96 [0.94–0.97]), and a personal doctor (0.96 [0.94–0.97]); however, this association was not observed among respondents with private insurance, individuals overdue for routine care, reporting cost barriers, or without a personal doctor (**S4 Table**; Fig 2)

**Table 4:** Subgroup analyses of the association between childhood abuse and cancer screening adherence, stratified by adult life socioeconomic and healthcare access status.

Effect Modifier	Subgroup	CRC Screening (IRR [95% CI], P-value)	Cervical Screening (IRR [95% CI], P-value)	Breast Screening (IRR [95% CI], P-value)
**Education**	<College	1.00 (0.98–1.01), p = 0.758	0.98 (0.96–1.00), p = 0.064	0.96 (0.94–0.98), p < **0.001**
	College graduate	1.01 (0.99–1.03), p = 0.218	0.96 (0.94–0.98), p < **0.001**	0.94 (0.92–0.96), p < **0.001**
**Income**	<$75,000	1.00 (0.98–1.01), p = 0.903	0.97 (0.95–0.99), p = **0.002**	0.95 (0.93–0.96), p < **0.001**
	≥ $75,000	1.02 (1.00–1.03), p = 0.079	0.97 (0.96–0.99), p = **0.003**	0.96 (0.94–0.98), p **< 0.001**
**Employment**	Unemp/Retired/Student	1.00 (0.99–1.01), p = 0.954	0.99 (0.96–1.03), p = 0.706	0.95 (0.93–0.97), p **< 0.001**
	Homemaker	0.99 (0.92–1.06), p = 0.758	0.96 (0.92–1.00), p = 0.068	0.96 (0.90–1.02), p = 0.155
	Employed	1.01 (0.99–1.03), p = 0.491	0.97 (0.95–0.98), p **< 0.001**	0.96 (0.94–0.98), p **< 0.001**
**Marital status**	Never married	1.02 (0.97–1.06), p = 0.472	0.99 (0.96–1.02), p = 0.509	0.96 (0.92–1.00), p = 0.059
	Div/Wid/Sep	0.98 (0.96–1.00), p = **0.035**	0.95 (0.92–0.98), p = **0.001**	0.92 (0.90–0.95), p **< 0.001**
	Married	1.01 (1.00–1.03), p = **0.043**	0.97 (0.96–0.99), p = **0.001**	0.97 (0.95–0.98), p **< 0.001**
**Home ownership**	Rented/Other	1.01 (0.98–1.05), p = 0.411	1.00 (0.98–1.03), p = 0.908	0.94 (0.91–0.98), p = **0.001**
	Own home	1.00 (0.99–1.01), p = 0.727	0.96 (0.95–0.98), p **< 0.001**	0.96 (0.94–0.97), p **< 0.001**

## Discussion

Childhood abuse has been associated with increased cancer risk, including cervical cancer, through mechanisms such as sexually transmitted infections, risky sexual behaviors, unhealthy lifestyle patterns, and reduced healthcare-seeking in adulthood [4,5,14]. Prior research suggests that survivors may be less likely to adhere to breast and cervical cancer screening guidelines, which could contribute to delayed detection [6]. Kaminga et al. reported 23%–26% higher odds of any cancer in adulthood among individuals with a history of physical or sexual abuse [4]. Less is known, however, about how adult life experiences and healthcare access are related to the abuse–screening relationship. In this nationally representative, cross-sectional analysis, we examined whether socioeconomic status and healthcare access were associated with differences in screening adherence among adults with a history of childhood abuse. These findings should be interpreted as associations rather than causal effects, but the findings do highlight potentially modifiable structural and access-related factors that may help identify groups at risk for under-screening and inform future targeted interventions and prospective studies to improve cancer screening uptake among adults with childhood adversity.

ACEs exert life-long effects on socioeconomic position, health behaviors, and access to healthcare [22,23]. Downing et al. reported 50% higher odds of poor general health and more frequent physical and mental unhealthy days among adults with a history of childhood abuse [3]. In a national survey of cervical cancer screening, Prather et al. reported 29% higher odds of Pap testing among women without abuse compared with individuals with abuse histories. Similarly, Alcala et al. [8] reported increased risk of CRC screening non-compliance in men with a history of abuse, while Kraft et al. [23] noted higher rates of breast cancer screening noncompliance among adults reporting ACEs. Building on this evidence, the current study examined the modifying role of adult life socioeconomic status and healthcare access on the relation of abuse-screening. Interestingly, the association between childhood abuse and screening adherence was generally similar across strata defined by education, income, and marital status, but appeared slightly more pronounced in some groups with relatively higher access or stability (e.g., employed and homeowners) compared with their disadvantaged counterparts. For breast cancer, survivors of abuse had lower screening adherence in multiple strata, including individuals with less than a college education, unemployed status, lower income, and divorced/widowed/separated status. One plausible explanation is a “*floor effect*”: disadvantaged groups already demonstrated lower baseline screening adherence in the risk-factor models regardless of abuse status, leaving less room for additional relative differences by abuse history. In contrast, among groups with better access and healthcare contact, screening adherence was generally higher, and a history of abuse may remain a salient barrier, potentially reflecting trauma-related care avoidance, lower trust, or other unmeasured psychosocial factors and resulting in a more detectable incremental reduction even when structural barriers are less prominent. Alternative explanations should also be considered. Because screening and abuse history were self-reported, measurement limitations (including differential recall or reporting of screening by healthcare engagement) could have contributed to apparent subgroup contrasts. In addition, selection/conditioning effects were possible; stratifying by markers of healthcare engagement or stability (factors that may be influenced by childhood abuse and are closely related to screening) may have introduced selection bias and yielded counterintuitive patterns. Together, these findings align with prior work linking socioeconomic disadvantage to reduced screening uptake and suggest that both structural barriers and trauma-related factors may contribute to persistent screening gaps among survivors of childhood abuse [24].

Notably, certain adult life experiences typically considered protective did not fully buffer the association between childhood abuse and reduced screening adherence. Although higher education was linked with modestly better overall outcomes, survivors with a college degree still demonstrated 4–6% lower screening adherence compared with their counterparts with no history of abuse (cervical: aIRR 0.96; breast: aIRR 0.94). Employment, often viewed as a marker of stability [25], appeared to exacerbate the negative association, whereas homemaking did not demonstrate an effect — potentially reflecting the protective value of reduced occupational stress. Marriage, another factor generally associated with health benefits [26,27], similarly failed to mitigate the influence of childhood abuse, as married survivors had lower screening adherence across cervical and breast cancer screening. These findings contrast with prior work from Hanske et al. who reported higher cancer screening compliance for colorectal (63%), cervical (29%), and breast (42%) cancers among married adults [28]. Taken together, the data suggest that while conferring some resilience, positive adult life milestones were insufficient to counterbalance the enduring effects of early life adversities. Psychological trauma from childhood abuse, especially sexual abuse, may directly heighten cancer risk through reduced engagement with preventive care. These findings underscore the need for early psychological support and targeted interventions to improve long-term physical, mental, and sexual health, alongside structural efforts to reduce disparities in preventive healthcare for survivors of childhood abuse.

Healthcare access barriers are well established as predictors of lower cancer screening adherence. Zhao et al reported that uninsured individuals were 41% less likely to undergo breast cancer screening, 19% less likely to receive cervical cancer screening, and 47–52% less likely to complete colorectal cancer screening [29]. In the current study, stratified analyses demonstrated that healthcare access modified the abuse–screening relationship; among the uninsured, childhood abuse was associated with lower breast cancer screening adherence (aIRR 0.86), although associations with CRC and cervical screening in this stratum did not reach statistical significance. These findings were consistent with prior research demonstrating that insurance coverage was a key predictor of improved adherence to both cancer and non-cancer preventive services [30,31]. These findings may reflect the higher likelihood of socioeconomic disadvantage among abuse survivors but also indicate that such individuals are particularly vulnerable to reduced healthcare-seeking behaviors and disparities in access.

Childhood abuse is an unchangeable past experience, but its long-term health consequences can possibly be addressed. Importantly, greater healthcare access did not fully eliminate abuse-related disparities in screening, highlighting the need to identify individuals with abuse histories, actively assess barriers to care, and provide targeted supports such as outreach, counseling, navigation, and subsidized preventive services [32,33]. Further prospective research is needed to clarify whether these associations are causal or partly reflect unmeasured confounding. Notably, for cervical and breast cancer screening, several factors typically considered favorable (e.g., having a regular checkup, a personal doctor, or no reported cost barrier) were associated with larger differences by abuse status, suggesting that trauma-related barriers may persist even among individuals engaged with care. These findings underscore the importance of training primary care clinicians in trauma-informed approaches and in recognizing how childhood abuse can shape preventive care behaviors [34]. While adherence to standardized cancer screening guidelines remains critical, additional flexibility and tailored interventions may be necessary to ensure equitable access among survivors of childhood abuse.

The current study benefits from the use of a large, nationally representative dataset, the BRFSS, with survey weighting applied to enhance generalizability to the U.S. adult population. A broad set of demographic, socioeconomic, healthcare access, and household challenge variables were included, allowing for a comprehensive assessment of the factors influencing the relationship between childhood abuse and cancer screening. Despite its strengths, the study findings should be interpreted with several limitations in mind. This was a cross-sectional analysis of survey-based data and is therefore subject to a few inherent limitations including data entry errors and systemic biases. Since the data depended on voluntary participation in the study and self-reported measures of abuse and screening, our results might be vulnerable to recall, social desirability, and non-response bias. The survey was conducted by telephone, which may underrepresent severely disadvantaged populations without reliable phone access. Interviews were administered by numerous surveyors, raising the possibility of variability in phrasing or interpretation. Age was reported in 5-year increments starting with the age group **18–24 years**, limiting inclusion of women aged 21–24 years in the cervical cancer cohort. Cervical and breast cancer screening analyses were restricted to eligible women according to USPSTF guidelines, which was necessary to define guideline-concordant adherence; nevertheless, the possibility of limitations arising from unmeasured differences in the prevalence of certain underlying factors cannot be excluded. While more recent data were available in BRFSS, based on the inclusion and exclusion criteria for the current study, only 2020 BRFSS database had sufficient sample size with data on history of childhood abuse as well as cancer screening. In addition, income was reported as total annual household income and could not be analyzed at the individual level. Subgroup (stratified) analyses also involved multiple comparisons and some strata likely had smaller sample sizes with wider confidence intervals; therefore, subgroup findings should be interpreted as exploratory and hypothesis-generating; conditioning on healthcare engagement variables (e.g., having a regular checkup, a personal doctor, or no cost barrier) may have introduced selection/conditioning bias that could contribute to unexpected subgroup patterns.

## Conclusion

In this nationally representative study, childhood abuse was associated with modestly lower adherence to colorectal, cervical, and breast cancer screening guidelines in fully adjusted models. Screening shortfalls were most consistent for cervical and breast screening across multiple socioeconomic and healthcare-access strata and were particularly pronounced among breast screening among uninsured adults. These findings underscore the importance of reducing structural barriers and expanding access to preventive care, alongside trauma-informed approaches, to improve cancer screening uptake among populations affected by childhood adversity.

## Supporting information

S1 File STROBE checklist. STROBE checklist for cross-sectional studies. The STROBE checklist is licensed under the Creative Commons Attribution 4.0 International License (CC BY 4.0).
(DOC)

S1 Table BRFSS variables used to operationalize eligibility. (DOCX)

S2 Table Baseline characteristics of respondents with and without a history of abuse. (DOCX)

S3 Table Complete adjusted models (Model 1) for Cancer screening adherence to guidelines for colorectal, cervical, and breast cancer. (DOCX)

S4 Table Subgroup Analyses of the Association Between Childhood Abuse and Cancer Screening Adherence, Stratified by Other Childhood Experiences and Adult Healthcare Access. (DOCX)
