# Supplementary material for: Past hurts, present hurdles: Analyzing the impact of adult socioeconomic status and healthcare access on cancer screening among childhood abuse survivors
Source: PLOS Glob Public Health. 2026 Aug 26;6(8):e0007073. doi: 10.1371/journal.pgph.0007073 (PMC13513954; doi:10.1371/journal.pgph.0007073)
Supplement: Supplementary file 1 [file pgph.0007073.s002.docx]

**S1 Table.** BRFSS variables used to operationalize eligibility

|  | **Screening Eligibility** |
| --- | --- |
| **Colorectal cancer** | - Age 50–74: _AGEG5YR = 7–11 - Exclude if prior history of CRC: CNCRTYP1 = 10 (Colon) OR 14 (Rectal) |
| **Cervical cancer** | - Age 25–64: _AGEG5YR = 2–9 - Female sex: SEXVAR = 2 - Exclude if prior cervical cancer: CNCRTYP1 = 2 (Cervical) - Exclude if hysterectomy: HADHYST2 = 1 (Yes) |
| **Breast cancer** | - Age 40–74: _AGEG5YR = 5–11 - Female sex: SEXVAR = 2 - Exclude if prior breast cancer: CNCRTYP1 = 1 (Breast) |
|  |  |
|  | **Screening Adherent if** |
| **Colorectal cancer** | - Colonoscopy within past 10 years (COLNSCPY=1 + COLNTEST=1—4), **OR** - Age 50-74 and had colonoscopy within past 10 years (_CLNSCPY =1), **OR** - Sigmoidoscopy within past 5 years (SIGMSCPY=1 + SIGMTEST=1—3), **OR** - BLDSTOL1=1 + LSTBLDS4=1 (i.e., FOBT within past 1 year); **OR** - Stool DNA test within past 1–3 years (STOOLDNA=1 + SDNATEST=1—3), **OR** - CT colonography within past 5 years (VIRCOLON=1 + VCLNTEST =1—4) |
| **Cervical cancer** | - Last Pap within prior 3 years and age 25-64 years (HADPAP2 =1 + LASTPAP2 = 1—3 + _AGEG5YR = 2—9), **OR** - Last HPV test within prior 5years and Age 30-64 years: HPVTEST =1 + HPLSTTST = 1—4 + _AGEG5YR = 3—9 |
| **Breast cancer** | - Mammography within past 2 years (HADMAM=Yes **+** HOWLONG = 1—2) |
| Note: _AGEG5YR is BRFSS 5-year age grouping; SEXVAR = 2 indicates female respondents. CNCRTYP1 identifies cancer type among respondents reporting a cancer history; values used here exclude site-specific prior cancers relevant to each screening outcome. For CRC adherence, _CLNSCPY is a BRFSS-derived indicator; primary definitions are also provided using the underlying test and timing variables. | |
