# Supplementary material for: Past hurts, present hurdles: Analyzing the impact of adult socioeconomic status and healthcare access on cancer screening among childhood abuse survivors
Source: PLOS Glob Public Health. 2026 Aug 26;6(8):e0007073. doi: 10.1371/journal.pgph.0007073 (PMC13513954; doi:10.1371/journal.pgph.0007073)
Supplement: Supplementary file 2 [file pgph.0007073.s003.docx]

**S2 Table.** Baseline characteristics of respondents with and without a history of abuse

| **Variable** | **Total** | **Non-abuse** | **Abuse** | **p-value** |
| --- | --- | --- | --- | --- |
| **History of cancer** |  |  |  |  |
| Any skin cancer history | 12,828 (7.0%) | 7,654 (7.5%) | 5,174 (6.5%) | **<0.001** |
| Any other cancer history | 11,719 (6.9%) | 6,701 (6.9%) | 5,018 (6.8%) | 0.055 |
| Any cancer history | 21,614 (12.4%) | 12,623 (12.8%) | 8,991 (11.9%) | <0.001 |
| CRC history | 462 (0.2%) | 288 (0.2%) | 174 (0.2%) | 0.009 |
| Cervical cancer history | 382 (0.2%) | 149 (0.1%) | 233 (0.3%) | <0.001 |
| Breast cancer history | 1,559 (0.6%) | 936 (0.7%) | 623 (0.6%) | 0.003 |
| **Abuse type** |  |  |  |  |
| Emotional abuse | 39,783 (33.9%) | 0 (0.0%) | 39,783 (75.1%) | <0.001 |
| Physical abuse | 27,944 (24.6%) | 0 (0.0%) | 27,944 (54.3%) | <0.001 |
| Sexual abuse | 15,948 (13.6%) | 0 (0.0%) | 15,948 (30.1%) | <0.001 |
| **Other Household challenges** |  |  |  |  |
| Combined | 58,334 (53.2%) | 22,066 (36.6%) | 36,268 (72.9%) | <0.001 |
| Alcoholic household | 27,415 (23.2%) | 8,021 (11.1%) | 19,394 (37.9%) | <0.001 |
| Divorced parents | 31,101 (31.5%) | 12,122 (22.6%) | 18,979 (42.3%) | <0.001 |
| Domestic violence | 18,844 (17.4%) | 3,143 (5.0%) | 15,701 (32.5%) | <0.001 |
| Drug abuse in household | 11,909 (11.9%) | 2,469 (4.2%) | 9,440 (21.1%) | <0.001 |
| Jail/prison household | 9,030 (9.7%) | 2,178 (4.1%) | 6,852 (16.5%) | <0.001 |
| Mentally ill household | 20,094 (17.3%) | 4,671 (6.9%) | 15,423 (29.9%) | <0.001 |
| **Cost barrier to care** | 11,285 (12.5%) | 4,281 (8.6%) | 7,004 (17.3%) | <0.001 |
| **Personal doctor** | 100,698 (75.6%) | 57,892 (77.3%) | 42,806 (73.5%) | <0.001 |
| **Routine checkup** |  |  |  | <0.001 |
| 5+ years/never | 6,784 (6.7%) | 3,253 (5.4%) | 3,531 (8.4%) |  |
| Within 2 years | 108,962 (87.2%) | 62,374 (89.3%) | 46,588 (84.6%) |  |
| Within 2–5 years | 6,118 (6.1%) | 3,031 (5.3%) | 3,087 (7.1%) |  |
| **Self-reported health** |  |  |  | <0.001 |
| Poor | 5,284 (4.0%) | 2,362 (3.0%) | 2,922 (5.2%) |  |
| Fair | 15,194 (12.3%) | 7,549 (10.5%) | 7,645 (14.5%) |  |
| Good | 37,617 (31.5%) | 20,848 (31.4%) | 16,769 (31.5%) |  |
| Very good | 41,690 (32.7%) | 24,049 (33.0%) | 17,641 (32.4%) |  |
| Excellent | 23,168 (19.5%) | 14,470 (22.1%) | 8,698 (16.4%) |  |
| **Rural residence** | 22,748 (9.8%) | 13,727 (10.8%) | 9,021 (8.5%) | <0.001 |
| **Metropolitan residence** | 78,722 (80.9%) | 43,013 (79.4%) | 35,709 (82.6%) | <0.001 |
| *All data are presented as sample frequencies and weighted percentages**.** | | | | |
