# Supplementary material for: Past hurts, present hurdles: Analyzing the impact of adult socioeconomic status and healthcare access on cancer screening among childhood abuse survivors
Source: PLOS Glob Public Health. 2026 Aug 26;6(8):e0007073. doi: 10.1371/journal.pgph.0007073 (PMC13513954; doi:10.1371/journal.pgph.0007073)
Supplement: Supplementary file 3 [file pgph.0007073.s004.docx]

**S3 Table**. Complete adjusted models (Model 1) for Cancer screening adherence to guidelines for colorectal, cervical, and breast cancer

| **Variable** | **CRC** | **Cervical** | **Breast** |
| --- | --- | --- | --- |
| No-abuse (ref.) | Ref. | Ref. | Ref. |
| **Childhood abuse** | 0.97 (0.94–1.00), p=0.022 | 0.97 (0.94–0.99), p=0.013 | 0.96 (0.93–0.99), p=0.005 |
| **HHC** (ref. No HHC) | 1.03 (1.00–1.06), p=0.042 | 1.01 (0.98–1.04), p=0.427 | 0.98 (0.95–1.01), p=0.151 |
| **Any cancer history** | 1.07 (1.04–1.11), p<0.001 | 1.06 (1.02–1.11), p=0.005 | 1.05 (1.01–1.08), p=0.006 |
| **Age groups** |  |  |  |
| Age 35–44 | NA | 0.93 (0.89–0.97), p<0.001 | NA |
| Age 45–54 | NA | 0.91 (0.87–0.95), p<0.001 | 1.40 (1.30–1.51), p<0.001 |
| Age 55–64 | 1.37 (1.30–1.44), p<0.001 | 0.88 (0.85–0.92), p<0.001 | 1.52 (1.41–1.64), p<0.001 |
| Age 65–74 | 1.44 (1.36–1.51), p<0.001 | NA | 1.55 (1.43–1.67), p<0.001 |
| **Race** (Ref: White) |  |  |  |
| Minority race | 1.06 (1.02–1.10), p=0.001 | 1.05 (1.02–1.08), p=0.002 | 1.16 (1.12–1.20), p<0.001 |
| Education (Ref: less than college) |  |  |  |
| College grad | 1.06 (1.03–1.09), p<0.001 | 1.04 (1.02–1.07), p=0.001 | 1.06 (1.02–1.09), p=0.001 |
| **Marital status** (Ref: never married) |  |  |  |
| Div/Wid/Sep | 1.01 (0.95–1.07), p=0.726 | 1.03 (0.99–1.09), p=0.168 | 0.96 (0.91–1.01), p=0.146 |
| Married | 1.12 (1.06–1.18), p<0.001 | 1.05 (1.02–1.09), p=0.006 | 1.07 (1.01–1.13), p=0.017 |
| **Employment** (Ref: unemployed) |  |  |  |
| Employed | 0.89 (0.86–0.92), p<0.001 | 1.04 (1.00–1.08), p=0.076 | 0.98 (0.94–1.01), p=0.234 |
| **Insurance** (Ref: uninsured) |  |  |  |
| Public | 1.96 (1.74–2.20), p<0.001 | 1.32 (1.22–1.43), p<0.001 | 1.73 (1.52–1.97), p<0.001 |
| Private | 1.83 (1.62–2.06), p<0.001 | 1.32 (1.23–1.41), p<0.001 | 1.77 (1.56–2.00), p<0.001 |
| **Income** (Ref: low income) |  |  |  |
| Higher income | 1.07 (1.04–1.11), p<0.001 | 1.06 (1.03–1.09), p<0.001 | 1.06 (1.03–1.10), p=0.001 |
| Rural (Ref: urban) | 0.96 (0.93–0.99), p=0.006 | 0.96 (0.93–1.00), p=0.039 | 0.97 (0.93–1.00), p=0.081 |

*Survey design details: CRC models included 12,995 observations (population size 10.87 million), 631 strata, and 12,995 PSUs, with 12,364 design degrees of freedom; model fit F(14,12351)=39.02, *p*<0.001. Cervical models included 6,993 observations (population size 7.23 million), 553 strata, and 6,993 PSUs, with 6,440 design degrees of freedom; model fit F(15,6426)=9.09, *p*<0.001. Breast models included 8,029 observations (population size 6.77 million), 575 strata, and 8,029 PSUs, with 7,454 design degrees of freedom; model fit F(15,7440)=15.99, *p*<0.001.
Abbreviations: CRC, colorectal cancer; HHC, household challenges; IRR, incidence rate ratio; Div/Wid/Sep, divorced/widowed/separated. All values reported as IRR [95% CI], P-value.
