# Supplementary material for: Past hurts, present hurdles: Analyzing the impact of adult socioeconomic status and healthcare access on cancer screening among childhood abuse survivors
Source: PLOS Glob Public Health. 2026 Aug 26;6(8):e0007073. doi: 10.1371/journal.pgph.0007073 (PMC13513954; doi:10.1371/journal.pgph.0007073)
Supplement: Supplementary file 4 [file pgph.0007073.s005.docx]

| **Effect Modifier** | **CRC Screening** | **Cervical Screening** | **Breast Screening** |
| --- | --- | --- | --- |
| **Household challenges** |  |  |  |
| No | 1.01 (0.99–1.03), p=0.200 | 0.97 (0.95–0.99), p=**0.002** | 0.96 (0.94–0.98), p**<0.001** |
| Yes | 0.99 (0.98–1.01), p=0.497 | 0.97 (0.96–0.99), p=**0.001** | 0.94 (0.93–0.96), p**<0.001** |
| **Insurance status** |  |  |  |
| Uninsured | 0.97 (0.89–1.06), p=0.515 | 0.97 (0.91–1.02), p=0.229 | 0.86 (0.78–0.95), p=**0.003** |
| Public | 0.99 (0.96–1.03), p=0.752 | 0.95 (0.89–1.03), p=0.203 | 0.95 (0.91–1.00), p=**0.032** |
| Private | 1.00 (0.96–1.05), p=0.843 | 0.97 (0.94–1.01), p=0.114 | 0.97 (0.92–1.01), p=0.118 |
| **Routine checkup** |  |  |  |
| ≥2 years/never | 1.11 (1.01–1.23), p=**0.037** | 0.98 (0.90–1.07), p=0.703 | 1.06 (0.89–1.26), p=0.493 |
| Within 2 years | 1.00 (0.99–1.01), p=0.710 | 0.98 (0.97–0.99), p=**0.001** | 0.96 (0.95–0.97), p**<0.001** |
| **Cost barrier** |  |  |  |
| No | 1.00 (0.99–1.02), p=0.504 | 0.97 (0.96–0.99), **p<0.001** | 0.96 (0.94–0.97), p**<0.001** |
| Yes | 1.02 (0.97–1.08), p=0.449 | 0.99 (0.95–1.04), p=0.821 | 0.97 (0.91–1.04), p=0.369 |
| **Personal doctor** |  |  |  |
| No | 1.05 (0.98–1.12), p=0.144 | 0.99 (0.95–1.04), p=0.703 | 0.95 (0.88–1.03), p=0.255 |
| Yes | 1.00 (0.99–1.01), p=0.769 | 0.97 (0.96–0.98), p**<0.001** | 0.96 (0.94–0.97), p**<0.001** |

**S4 Table.** Subgroup Analyses of the Association Between Childhood Abuse and Cancer Screening Adherence, Stratified by Other Childhood Experiences and Adult Healthcare Access.

Significant p-values were defined as p<0.05. All values reported as IRR [95% CI], P-value.
Abbreviations: IRR, incidence rate ratio.
