# Supplementary material for: Past hurts, present hurdles: Analyzing the impact of adult socioeconomic status and healthcare access on cancer screening among childhood abuse survivors
Source: PLOS Glob Public Health. 2026 Aug 26;6(8):e0007073. doi: 10.1371/journal.pgph.0007073 (PMC13513954; doi:10.1371/journal.pgph.0007073)
Supplement: Supplementary file 5 [file pgph.0007073.s007.docx]

**Catherine Kyobutungi**
Editor-in-Chief
PLOS Global Public Health

February 22, 2026

Re: PGPH-D-25-04092 **“Past hurts, present hurdles: Analyzing the impact of adult life experiences on cancer screening among childhood abuse survivors”**

Dear Dr. Kyobutungi,

We are pleased that our manuscript was reviewed and were invited for resubmission for publication pending revisions.

We thank the reviewers for their valuable insight and comments as these serve to further strengthen our manuscript.

As requested, we have provided a point-by-point response to each of the reviewers’ comments with relevant changes made to the manuscript.

**Comments from the Editors and Reviewers:**

 Journal Requirements:

1. *Please ensure that all Figure files have corresponding citations and legends within the manuscript. Currently, Figure 1 & 2 in your submission file inventory does not have an in-text citation. If the figure is no longer to be included as part of the submission, please remove it from the file inventory.*

*If the reviewer comments include a recommendation to cite specific previously published works, please review and evaluate these publications to determine whether they are relevant and should be cited. There is no requirement to cite these works unless the editor has indicated otherwise.* 

**Response to Editor:** Thank you for your time and consideration of our manuscript for publication in your esteemed journal. Figure 1 and 2 have now been appropriately cited in the main text (**Fig 1 and Fig 2**).

We have also reviewed and revised all our references as appropriate and as suggested by the reviewer.

**Results, Line 281	Page 14**

**Results, Line	289	Page 15**

**Review Comments to the Author**

**Reviewer #1:** This article addresses an important and current issue by examining how childhood abuse relates to cancer screening within the context of social and health service inequalities. However, my suggestions for the study are as follows:

**Title**:

1. *The title generally reflects the subject of the study; however, the phrase “adult life experiences” is quite broad. Since the text primarily addresses socioeconomic status and access to healthcare, a clearer expression of these two dimensions in the title could clarify the focus of the study.*

**Response to reviewer:** We thank the reviewer for their time and suggestions Their review helps us immensely improve our work. We agree with the reviewer and have revised the title to more precisely reflect these dimensions and better align with the study aims.

**Revised title:**
“Past hurts, present hurdles: Analyzing the impact of adult socioeconomic status and healthcare access on cancer screening among childhood abuse survivors”

**Abstract**:

1. *The abstract clearly presents the aim and main findings of the study; however, it is not entirely consistent with the definitions in the methods section. The age ranges and inclusion criteria given for cervical and breast cancer screening appear inconsistent between the abstract and the main text. The statement in the conclusion that “childhood abuse is not independently associated” is overly definitive, considering the structure of the adjusted models used. It should be more cautiously stated that this statement reflects the findings after the inclusion of access and socioeconomic variables.*

**Response to reviewer:** We thank the reviewer for these helpful comments. We revised the Abstract to align precisely with the Methods definitions by explicitly stating the BRFSS-adapted eligibility criteria for screening cohorts (CRC 50–74 years; cervical screening women 25–64 years, excluding hysterectomy; breast screening women 40–74 years) and associated inclusion/exclusion criteria. We also revised the Abstract conclusion to avoid the definitive statements that childhood abuse is “not independently associated,” and instead clarify that estimates are sensitive to covariate adjustment, particularly after inclusion of socioeconomic and healthcare access variables, consistent with the structure of our adjusted models.

**Abstract, Lines 20-48	Page 2-3**

**Introduction**:

1. *The introduction generally summarizes the importance of the topic and the existing literature well. The study's unique contribution can be more clearly highlighted as examining how access and socioeconomic conditions alter this relationship.*

**Response to reviewer:** We thank the reviewer for their thoughtful suggestion. We agree that the manuscript’s unique contribution can be stated more explicitly including effects of adult socioeconomic conditions and healthcare access on the association between childhood abuse and adherence to cancer screening. We have revised the Introduction to more clearly emphasize this novelty and to frame access and socioeconomic conditions as key contextual factors shaping screening disparities among survivors.

**Introduction, Lines 83-95	Pages 4-5**

**Methods**:

1. *While eligibility for cervical cancer screening is stated as 25–65 years, the definition of eligibility includes the phrase "Pap test for the 21–30 age group." This is inconsistent both internally and with USPSTF recommendations.*

**Response to reviewer:** We agree and have corrected this inconsistency. USPSTF recommends cervical cancer screening starting at age 21 (Pap smear every 3 years for ages 21–29; for ages 30–65, either Pap smear every 3 years, high-risk HPV testing every 5 years, or co-testing every 5 years). However, in BRFSS, age is not available as a continuous variable but as 5-year age categories (i.e., 18–24, 25–29, 30–34, etc.). Because BRFSS does not allow us to isolate ages 21–24 from the broader 18–24 category, we cannot precisely apply guideline eligibility beginning at age 21 without misclassifying women aged 18–20 as eligible. To avoid this, we defined the cervical screening-eligible cohort starting at the first age band that aligns with USPSTF eligibility without including ineligible ages (i.e., ≥25 years in BRFSS age groups) and revised the Methods accordingly. We also added this point as a limitation in the Discussion.

**Methods, Lines 119-134	Page 6**

**Methods, Lines 144-155	Page 7**

**Discussion, Lines 386-398	Page 20**

2. *Breast cancer screening: The association of the 35–65 age range with the 2016 USPSTF guidelines seems to require clarification.*

**Response to reviewer:** Thank you for pointing out this typographical error. Breast cancer screening eligibility in our primary analysis was females aged 40–74 years, consistent with the 2016 USPSTF recommendations. We have corrected this in the Methods and throughout the manuscript. The 2016 USPSTF guideline recommended biennial mammography for women aged 50–74 years, and advised that screening for women aged 40–49 years should be an individualized decision based on patient–clinician discussion. To align eligibility with the guideline’s age range that includes individualized screening for ages 40–49, and to ensure adequate sample size for stratified analyses, we defined eligibility as 40–74 years in the primary model. As a sensitivity analysis, we repeated the breast screening models restricting eligibility to women aged 50–74 years and noted consistent results. These corrections have been incorporated in the Methods, Results, and Table 3.

**Methods, Lines 129-134	Page 6**

**Results, Lines 250-252	Page 13**

**Table 3**

3. *The age ranges used for each type of screening should be clearly stated.*

**Response to reviewer:** We have now explicitly stated the **eligibility age ranges** used for each screening outcome in both the Methods based on USPSTF recommendations and adapted to BRFSS age categorization where applicable.

**Methods, Lines 119-134	Page 6**

4. *A detailed explanation of how the BRFSS variables are adapted to the guideline criteria should be provided.*

**Response to reviewer:** We thank the reviewer and agree. We have expanded the Methods and added detail in the Supplementary file to explicitly describe how BRFSS screening items were operationalized to reflect USPSTF guideline-based eligibility and up-to-date screening (adherence) for colorectal, cervical, and breast cancer screening. Specifically, we mapped BRFSS items capturing time since the most recent test (and, where applicable, test type) to USPSTF-recommended screening intervals. We also clarified outcome-specific exclusions using BRFSS history items, including self-reported prior cancer diagnoses (CRC, cervical, or breast cancer, as applicable) and hysterectomy for cervical cancer screening eligibility/adherence. The full derivation of each screening outcome from BRFSS variables (including item names/response categories and analytic cut-points) is provided in **Table S1**.

**Methods, Lines 144-155	Page 7**

**Table S1**

**Findings:**

1. *There are some numerical and directional inconsistencies between the text and the tables (e.g., personal physician ownership and insurance distributions). Text-table consistency, especially in Table 1, should be reviewed.*

**Response to reviewer:** Thank you for pointing out these inconsistencies. We have now revised all our results with accurate and consistent reporting.

**Results, Lines 212-294	Pages 12-15**

**Table 1-4 and Table S2-25**

5. *Typographical errors, such as the way population size is expressed, impair reliability.*

**Response to reviewer:** The manuscript has been revised to remove any typographical errors.

**Results, Line 213	Page 10**

6. *When interpreting subgroup findings, the impact of multiple test probability and sample sizes should be addressed more clearly.*

**Response to reviewer:**

**Methods, Lines 197-201	Page 9**

**Results, Lines 272-286	Page 14**

**Discussion, Lines 401-406	Page 20**

**Discussion:**

1. *The discussion section is strong in establishing a connection with the literature; however, more caution should be exercised in the causal interpretation of the results.*

**Response to reviewer:** We agree and have strengthened the Discussion to avoid causal language. We now explicitly frame findings as associations from a cross-sectional, self-reported survey, emphasize the potential for residual confounding and reverse causation, and clarify that effect-modification results are exploratory and interpreted primarily by effect sizes and confidence intervals rather than causal inference.

**Discussion, Lines 297-406	Page 16-20**

7. *Alternative explanations (measurement limitations, selection effect, base effect, etc.) should be evaluated in more detail, especially for unexpected findings seen in the “those without cost barriers” or “those with regular doctor” groups.*

**Response to reviewer:** We agree and expanded the Discussion (Lines 318–338, Pages 17–18) to address alternative explanations for the unexpected patterns in the no cost barrier and personal/regular doctor strata. We now discuss a base/floor effect (very low baseline screening among those with major access barriers), measurement limitations of self-reported abuse and screening (including differential recall/reporting by healthcare engagement), and potential selection/conditioning bias when stratifying by healthcare-engagement variables that may be downstream of childhood abuse. We also clarify that these subgroup findings are exploratory/hypothesis-generating and interpreted primarily by effect sizes and confidence intervals.

**Discussion, Lines 319-339	Page 17-18**

**Conclusion:**

1. *The results are valuable in highlighting the importance of access and socioeconomic conditions; however, instead of stating definitively that “there is no independent association,” it should be stated more balancedly that the findings are dependent on the adjustment strategy.*

**Response to reviewer:** We revised the Conclusion to avoid definitive causal language and to note that estimates are dependent on the adjustment strategy, particularly adjustment for socioeconomic and healthcare access factors, while preserving the main observed screening shortfalls (most consistently for cervical and breast screening).

**Conclusion, Lines 409-415	Page 21**

Thank you for considering our revised manuscript.

Timothy M. Pawlik, MD, PhD, MPH, MTS, MBA, FACS, FSSO,

MAMSE, FRACS (Hon.)

Professor and Chair, Department of Surgery

The Urban Meyer III and Shelley Meyer Chair for Cancer Research

Professor of Surgery, Oncology, and Health Services Management and Policy

Surgeon in Chief, The Ohio State University Wexner Medical Center

The Ohio State University, Wexner Medical Center

395 W. 12^th^ Ave., Suite 670

Columbus, OH 43210

614 293 8701 phone

614 293 4063 fax
