# Supplementary material for: Past hurts, present hurdles: Analyzing the impact of adult socioeconomic status and healthcare access on cancer screening among childhood abuse survivors
Source: PLOS Glob Public Health. 2026 Aug 26;6(8):e0007073. doi: 10.1371/journal.pgph.0007073 (PMC13513954; doi:10.1371/journal.pgph.0007073)
Supplement: Supplementary file 6 [file pgph.0007073.s008.docx]

**Catherine Kyobutungi**
Editor-in-Chief
PLOS Global Public Health

June 28^th^, 2026

Re: PGPH-D-25-04092 **“Past hurts, present hurdles: Analyzing the impact of adult life experiences on cancer screening among childhood abuse survivors”**

Dear Dr. Kyobutungi,

We are pleased that our manuscript was reviewed and was invited for resubmission for publication pending revisions.

We thank the reviewers for their valuable insight and comments as these serve to further strengthen our manuscript.

Below, we provide a point-by-point response to the Editor’s and Reviewer’s comments, along with the corresponding changes made to the manuscript.

**Comments from the Editors and Reviewers:

Reviewer 1:**

*- The manuscript seems well written and deals with an important issue.

- However, the ethical aspect of the study needs to be further revisited.

- Even though the claim that the study is “exempt from IRB review because it used secondary analysis and no identifiable private information” seems valid in principle in most institutional policies, I am afraid it may not be automatically exempt simply because it is secondary data analysis.

- The best thing to do would be that the protocol be submitted to an IRB/ethics committee and that IRB to determine whether it is exempt or eligible for expedited/full review. Issues such as childhood abuse/trauma, socioeconomic disadvantage, healthcare access, and cancer screening behaviors may be considered as highly sensitive topics, and ethics committee may still require to examine risks (of stigmatization or re-identification of certain groups) even when identifiers are removed.

Therefore, the exemption determination should be made by an IRB/ethics committee, not solely by investigators.*

**Response to comment:** We thank the Editor and Reviewer for this important comment. The Ohio State University Institutional Review Board reviewed the study protocol and determined the study as Exempted from IRB approval due to the secondary analysis of de-identified publicly available data used for the study. We have revised the Methods section of the manuscript to clearly state IRB exemption by the Ohio State University IRB along with the IRB ID # **STUDY20261166.

Materials and Methods, Page 7, Lines 143-147**

**Reviewer 2:**
*1) We note that only three types of cancer are analyzed (colorectal, breast and cervical cancer). Please provide justification for why other cancers were not examined. Please also describe how the eligibility criteria may affect your results. For example, eligibility criteria for breast and cervical cancer were restricted to women based on the manifestation of these cancers but are there other variables specific to women that may affect the results (i.e. higher reporting of ACES upon women or higher prevalence of ACES upon women)?*

**Response:** We thank the reviewer for this important comment. Overall, only four common cancers have strong recommendations for screening suggested by the United States Prevention and Screening Task Force (USPSTF) in the general population including CRC, lung, breast, and cervical cancers. Of these four, this study only focused on three cancers (CRC, cervical, and breast) excluding Lung cancer due to the very strict screening criteria for lung cancer that, when reinforced by other exclusion criteria for history of childhood abuse and data on chest CT, severely reduced the number of respondents available for the study. Specifically, the 2020 BRFSS Lung Cancer Screening questions were administered as an optional module and were available only in selected participating states (Delaware, Maine, New Jersey, North Dakota, South Dakota), which would have substantially restricted the analytic sample and limited generalizability.

Furthermore, while screening tests are available for some other cancers, they are only recommended in small groups of individuals rather than the general population. For instance, PSA-based screening for prostate cancer is based on individualized/shared decision-making in men over age 65years rather than a universal adherence-based screening recommendation. The relevant BRFSS content for prostate cancer screening was also administered only as an optional module with very limited sample size. (<https://www.uspreventiveservicestaskforce.org/uspstf/index.php/recommendation/prostate-cancer-screening>). These are the reasons why most studies on cancer screening include only these few cancer types and not others. We have revised the Methods to clarify this rationale.

Regarding eligibility, our inclusion and exclusion criteria very closely followed the USPSTF guidelines including restriction of cervical and breast cancer screening analyses to women only without a history of hysterectomy or the corresponding cancers. Moreover, while some studies may have reported differences in the rates of ACEs by gender, the overall sex distribution was similar between respondents with and without childhood abuse in our analytic cohort. Multivariable models also adjusted for demographic, socioeconomic, healthcare access, self-rated health, cancer history, and other childhood household challenge variables, further reducing potential confounding by measured factors.

Nevertheless, we agree that limitations arising from unmeasured differences in the prevalence of certain underlying factors cannot be excluded. We therefore revised our manuscript to include these limitations.

**Materials and Methods, Page 7, Lines 135-136**

**Discussion, Page 21, Lines 410-416**

*2) Please provide justification for using the 2020 Behavioral Risk Factor Surveillance System (BRFSS) instead of more recent versions of this data.*

**Response:** In BRFSS database, the ACE module is optional and highly varies by state participation by year. Based on CDC BRFSS module documentation, 28 jurisdictions administered the ACE module in 2020, compared with fewer jurisdictions in subsequent years (16 in 2021, 12 in 2022, 12 in 2023, 13 in 2024). For our study, we required the eligible respondents to answer questions on history of childhood abuse as well as on cancer screening. This strict criterion highly limited the number of eligible respondents. Only the 2020 BRFSS was selected because it provided the broadest availability of the ACE module along with cancer screening data. Adding data from more years dilutes the numbers of eligible sample, increases missingness as well as the risk of selection and non-response bias. Lastly, using a more recent single year would have substantially reduced the analytic sample and precision, while pooling multiple years would also have introduced heterogeneity due to changing state participation in the optional ACE module and possible differences in survey administration across years. We have clarified this rationale in the Methods.

**Materials and Methods, Page 7, Lines 137-139**

**Discussion, Page 21, Lines 413-416**

Thank you for reconsidering our revised manuscript.

**Timothy M. Pawlik, MD, PhD, MPH, MTS, MBA, FACS, FSSO,**

**MAMSE, FRACS (Hon.)**

Professor and Chair, Department of Surgery

The Urban Meyer III and Shelley Meyer Chair for Cancer Research

Professor of Surgery, Oncology, and Health Services Management and Policy

Surgeon in Chief, The Ohio State University Wexner Medical Center

The Ohio State University, Wexner Medical Center

395 W. 12^th^ Ave., Suite 670

Columbus, OH 43210

phone 614 293 8701

fax 614 293 4063
