# Supplementary material for: Past hurts, present hurdles: Analyzing the impact of adult socioeconomic status and healthcare access on cancer screening among childhood abuse survivors
Source: PLOS Glob Public Health. 2026 Aug 26;6(8):e0007073. doi: 10.1371/journal.pgph.0007073 (PMC13513954; doi:10.1371/journal.pgph.0007073)
Supplement: Peer review history

# Peer review: Decision Letter 0

**DOI:** 10.1371/journal.pgph.0007073.r001

**Authors:** Ilse Bloom

12 Feb 2026

PGPH-D-25-04092

Past hurts, present hurdles: Analyzing the impact of adult life experiences on cancer screening among childhood abuse survivors

PLOS Global Public Health

Dear Dr. Pawlik,

Thank you for submitting your manuscript to PLOS Global Public Health. After careful consideration, we feel that it has merit but does not fully meet PLOS Global Public Health’s publication criteria as it currently stands. Therefore, we invite you to submit a revised version of the manuscript that addresses the points raised during the review process.

Please note that we have only been able to secure a single reviewer to assess your manuscript. We are issuing a decision on your manuscript at this point to prevent further delays in the evaluation of your manuscript. Please be aware that the editor who handles your revised manuscript might find it necessary to invite additional reviewers to assess this work once the revised manuscript is submitted. However, we will aim to proceed on the basis of this single review if possible.

The comments from the reviewer can be found below. Please review these comments and make the appropriate revisions to your manuscript alongside a point-by-point response to the concerns raised.

Please submit your revised manuscript by Mar 29 2026 11:59PM. If you will need more time than this to complete your revisions, please reply to this message or contact the journal office at globalpubhealth@plos.org. When you're ready to submit your revision, log on to https://www.editorialmanager.com/pgph/ and select the 'Submissions Needing Revision' folder to locate your manuscript file.

Please include the following items when submitting your revised manuscript:

- A letter that responds to each point raised by the editor and reviewer(s). You should upload this letter as a separate file labeled 'Response to Reviewers'.
- A marked-up copy of your manuscript that highlights changes made to the original version. You should upload this as a separate file labeled 'Revised Manuscript with Track Changes'.
- An unmarked version of your revised paper without tracked changes. You should upload this as a separate file labeled 'Manuscript'.

Guidelines for resubmitting your figure files are available below the reviewer comments at the end of this letter.

We look forward to receiving your revised manuscript.

Kind regards,

Ilse Bloom

Staff Editor

PLOS Global Public Health

Journal Requirements:

1. Please ensure that all Figure files have corresponding citations and legends within the manuscript. Currently, Figure 1 & 2 in your submission file inventory does not have an in-text citation. If the figure is no longer to be included as part of the submission, please remove it from the file inventory.

If the reviewer comments include a recommendation to cite specific previously published works, please review and evaluate these publications to determine whether they are relevant and should be cited. There is no requirement to cite these works unless the editor has indicated otherwise.

Additional Editor Comments (if provided):

[Note: HTML markup is below. Please do not edit.]

Reviewers' comments:

Reviewer's Responses to Questions

**Comments to the Author**

1. Does this manuscript meet PLOS Global Public Health’s publication criteria? Is the manuscript technically sound, and do the data support the conclusions? The manuscript must describe methodologically and ethically rigorous research with conclusions that are appropriately drawn based on the data presented.

Reviewer #1: Partly

**********

2. Has the statistical analysis been performed appropriately and rigorously?

Reviewer #1: Yes

**********

3. Have the authors made all data underlying the findings in their manuscript fully available (please refer to the Data Availability Statement at the start of the manuscript PDF file)?

The PLOS Data policy requires authors to make all data underlying the findings described in their manuscript fully available without restriction, with rare exception. The data should be provided as part of the manuscript or its supporting information, or deposited to a public repository. For example, in addition to summary statistics, the data points behind means, medians and variance measures should be available. If there are restrictions on publicly sharing data—e.g. participant privacy or use of data from a third party—those must be specified.

Reviewer #1: Yes

**********

4. Is the manuscript presented in an intelligible fashion and written in standard English?

PLOS Global Public Health does not copyedit accepted manuscripts, so the language in submitted articles must be clear, correct, and unambiguous. Any typographical or grammatical errors should be corrected at revision, so please note any specific errors here.

Reviewer #1: Yes

**********

5. Review Comments to the Author

Please use the space provided to explain your answers to the questions above. You may also include additional comments for the author, including concerns about dual publication, research ethics, or publication ethics. (Please upload your review as an attachment if it exceeds 20,000 characters)

Reviewer #1: This article addresses an important and current issue by examining how childhood abuse relates to cancer screening within the context of social and health service inequalities. However, my suggestions for the study are as follows:

Title: The title generally reflects the subject of the study; however, the phrase “adult life experiences” is quite broad. Since the text primarily addresses socioeconomic status and access to healthcare, a clearer expression of these two dimensions in the title could clarify the focus of the study.

Abstract: The abstract clearly presents the aim and main findings of the study; however, it is not entirely consistent with the definitions in the methods section. The age ranges and inclusion criteria given for cervical and breast cancer screening appear inconsistent between the abstract and the main text. The statement in the conclusion that “childhood abuse is not independently associated” is overly definitive, considering the structure of the adjusted models used. It should be more cautiously stated that this statement reflects the findings after the inclusion of access and socioeconomic variables.

Introduction: The introduction generally summarizes the importance of the topic and the existing literature well. The study's unique contribution can be more clearly highlighted as examining how access and socioeconomic conditions alter this relationship.

Method: While eligibility for cervical cancer screening is stated as 25–65 years, the definition of eligibility includes the phrase "Pap test for the 21–30 age group." This is inconsistent both internally and with USPSTF recommendations.

Breast cancer screening: The association of the 35–65 age range with the 2016 USPSTF guidelines seems to require clarification.

The age ranges used for each type of screening should be clearly stated.

A detailed explanation of how the BRFSS variables are adapted to the guideline criteria should be provided.

Findings: There are some numerical and directional inconsistencies between the text and the tables (e.g., personal physician ownership and insurance distributions). Text-table consistency, especially in Table 1, should be reviewed.

Typographical errors, such as the way population size is expressed, impair reliability.

When interpreting subgroup findings, the impact of multiple test probability and sample sizes should be addressed more clearly.

Discussion: The discussion section is strong in establishing a connection with the literature; however, more caution should be exercised in the causal interpretation of the results.

Alternative explanations (measurement limitations, selection effect, base effect, etc.) should be evaluated in more detail, especially for unexpected findings seen in the “those without cost barriers” or “those with regular doctor” groups.

Conclusion: The results are valuable in highlighting the importance of access and socioeconomic conditions; however, instead of stating definitively that “there is no independent association,” it should be stated more balancedly that the findings are dependent on the adjustment strategy.

**********

6. PLOS authors have the option to publish the peer review history of their article (what does this mean?). If published, this will include your full peer review and any attached files.

**Do you want your identity to be public for this peer review?**  If you choose “no”, your identity will remain anonymous but your review may still be made public.

For information about this choice, including consent withdrawal, please see our Privacy Policy.

Reviewer #1: No

**********

[NOTE: If reviewer comments were submitted as an attachment file, they will be attached to this email and accessible via the submission site. Please log into your account, locate the manuscript record, and check for the action link "View Attachments". If this link does not appear, there are no attachment files.]

-->Figure Resubmissions:

While revising your submission, we strongly recommend that you use PLOS’s NAAS tool (https://ngplosjournals.pagemajik.ai/artanalysis) to test your figure files. NAAS can convert your figure files to the TIFF file type and meet basic requirements (such as print size, resolution), or provide you with a report on issues that do not meet our requirements and that NAAS cannot fix.

After uploading your figures to PLOS’s NAAS tool - https://ngplosjournals.pagemajik.ai/artanalysis, NAAS will process the files provided and display the results in the "Uploaded Files" section of the page as the processing is complete. If the uploaded figures meet our requirements (or NAAS is able to fix the files to meet our requirements), the figure will be marked as "fixed" above. If NAAS is unable to fix the files, a red "failed" label will appear above. When NAAS has confirmed that the figure files meet our requirements, please download the file via the download option, and include these NAAS processed figure files when submitting your revised manuscript.

# Author comment: Author response to Decision Letter 1

**DOI:** 10.1371/journal.pgph.0007073.r002

23 Feb 2026

Attachment Submitted filename: PGPH-D-25-04092 (R1) - Response to Reviewers.docx

# Peer review: Decision Letter 1

**DOI:** 10.1371/journal.pgph.0007073.r003

**Authors:** Damen Haile Mariam

25 May 2026

PGPH-D-25-04092R1

Past hurts, present hurdles: Analyzing the impact of adult socioeconomic status and healthcare access on cancer screening among childhood abuse survivors

PLOS Global Public Health

Dear Dr. Pawlik,

Thank you for submitting your manuscript to PLOS Global Public Health. After careful consideration, we have decided that your manuscript does not meet our criteria for publication and must therefore be rejected.

We are sorry that we cannot be more positive on this occasion. We very much appreciate your wish to present your work in one of PLOS's Open Access publications. Thank you for your support, and we hope that you will consider PLOS Global Public Health for other submissions in the future.

Yours sincerely,

Katherine Kokkinias, Ph.D.

Staff Editor, PLOS Global Public Health

Issued on behalf of

Damen Haile Mariam, MD, MPH, PhD

Academic Editor

PLOS Global Public Health

Additional Editor Comments (if provided):

General Comment -

- The manuscript seems well written and deals with an important issue.

- However, the ethical aspect of the study needs to be further revisited.

- Even though the claim that the study is “exempt from IRB review because it used secondary analysis and no identifiable private information” seems valid in principle in most institutional policies, I am afraid it may not be automatically exempt

simply because it is secondary data analysis.

- The best thing to do would be that the protocol be submitted to an IRB/ethics committee and that IRB to determine whether it is exempt or eligible for expedited/full review. Issues such as childhood abuse/trauma, socioeconomic disadvantage,

healthcare access, and cancer screening behaviors may be considered as highly sensitive topics, and ethics committee may still require to examine risks (of stigmatization or re-identification of certain groups) even when identifiers are removed.

Therefore, the exemption determination should be made by an IRB/ethics committee, not solely by investigators.

[Note: HTML markup is below. Please do not edit.]

Reviewers' comments:

Reviewer's Responses to Questions

**Comments to the Author**

1. If the authors have adequately addressed your comments raised in a previous round of review and you feel that this manuscript is now acceptable for publication, you may indicate that here to bypass the “Comments to the Author” section, enter your conflict of interest statement in the “Confidential to Editor” section, and submit your "Accept" recommendation.

Reviewer #1: All comments have been addressed

**********

2. Does this manuscript meet PLOS Global Public Health’s publication criteria? Is the manuscript technically sound, and do the data support the conclusions? The manuscript must describe methodologically and ethically rigorous research with conclusions that are appropriately drawn based on the data presented.

Reviewer #1: Yes

**********

3. Has the statistical analysis been performed appropriately and rigorously?

Reviewer #1: Yes

**********

4. Have the authors made all data underlying the findings in their manuscript fully available (please refer to the Data Availability Statement at the start of the manuscript PDF file)?

The PLOS Data policy requires authors to make all data underlying the findings described in their manuscript fully available without restriction, with rare exception. The data should be provided as part of the manuscript or its supporting information, or deposited to a public repository. For example, in addition to summary statistics, the data points behind means, medians and variance measures should be available. If there are restrictions on publicly sharing data—e.g. participant privacy or use of data from a third party—those must be specified.

Reviewer #1: Yes

**********

5. Is the manuscript presented in an intelligible fashion and written in standard English?

PLOS Global Public Health does not copyedit accepted manuscripts, so the language in submitted articles must be clear, correct, and unambiguous. Any typographical or grammatical errors should be corrected at revision, so please note any specific errors here.

Reviewer #1: Yes

**********

6. Review Comments to the Author

Please use the space provided to explain your answers to the questions above. You may also include additional comments for the author, including concerns about dual publication, research ethics, or publication ethics. (Please upload your review as an attachment if it exceeds 20,000 characters)

Reviewer #1: Thank you for addressing the suggested revisions. The title has been clarified, and the Methods section now more clearly describes the screening age ranges and how the BRFSS variables were aligned with guideline recommendations. In addition, the Discussion and Conclusion sections have been revised to adopt more cautious language regarding causal interpretations, and the limitations related to subgroup analyses have been more clearly acknowledged. These revisions have improved the clarity and overall reporting quality of the manuscript. I have no further comments.

**********

7. PLOS authors have the option to publish the peer review history of their article (what does this mean?). If published, this will include your full peer review and any attached files.

**Do you want your identity to be public for this peer review?**  If you choose “no”, your identity will remain anonymous but your review may still be made public.

For information about this choice, including consent withdrawal, please see our Privacy Policy.

Reviewer #1: No

**********

[NOTE: If reviewer comments were submitted as an attachment file, they will be attached to this email and accessible via the submission site. Please log into your account, locate the manuscript record, and check for the action link "View Attachments". If this link does not appear, there are no attachment files.]

# Author comment: Author response to Decision Letter 2

**DOI:** 10.1371/journal.pgph.0007073.r004

28 Jun 2026

Attachment Submitted filename: PGPH-D-25-04092 (R3)- Response.docx

# Editor report: Decision Letter 2

**DOI:** 10.1371/journal.pgph.0007073.r005

**Authors:** Jose Nazif-Munoz

22 Jul 2026

Past hurts, present hurdles: Analyzing the impact of adult socioeconomic status and healthcare access on cancer screening among childhood abuse survivors

PGPH-D-25-04092R2

Dear Dr. Kyobutungi and colleagues,

We are pleased to inform you that your manuscript, "Past hurts, present hurdles: Analyzing the impact of adult socioeconomic status and healthcare access on cancer screening among childhood abuse survivors" (PGPH-D-25-04092), has been provisionally accepted for publication in PLOS Global Public Health.

I have carefully considered the revised manuscript, the reviewers' comments, and your detailed point-by-point responses. I am pleased to note that you have addressed the reviewers' concerns comprehensively and thoughtfully. The revisions have improved the clarity and overall quality of the manuscript, particularly regarding the eligibility criteria for the screening outcomes, the interpretation of the adjusted analyses, and the presentation of the study's methodological approach. Your responses were clear, scientifically justified, and appropriately reflected in the revised manuscript.

In my assessment, the manuscript now satisfies the publication criteria of PLOS Global Public Health, and I am pleased to recommend it for publication.

Before your manuscript can be formally accepted, you will need to complete some formatting changes, which you will receive in a follow-up email. A member of the PLOS team will contact you with a set of requests.

Please note that your manuscript will not be scheduled for publication until the required changes have been completed, so a prompt response is appreciated.

IMPORTANT: The editorial review process is now complete. From this point onward, PLOS will only permit corrections related to spelling, formatting, or significant scientific errors. Requests for major changes, or changes that affect the scientific interpretation of your work, may result in delays to publication.

If your institution or institutions have a press office, please notify them about your upcoming publication to help maximize its impact. If press materials are being prepared, please inform the PLOS press team as soon as possible, and no later than 48 hours after receiving the formal acceptance. Your manuscript will remain under strict press embargo until 2 pm Eastern Time on the date of publication. For more information, please contact globalpubhealth@plos.org.

Thank you again for supporting Open Access publishing. We look forward to publishing your work in PLOS Global Public Health.

Congratulations on this important contribution.

Best regards,

José Ignacio Nazif-Muñoz, Ph.D.

Academic Editor

PLOS Global Public Health

Sincerely,

José Ignacio Nazif-Muñoz

Academic Editor

Reviewer Comments (if any, and for reference):
